# A Novel Water-Cut Sensing Method for a Multiphase-Flow Pipeline Using a Ridged-Horn Antenna

**DOI:** 10.3390/s26082466

**Published:** 2026-04-16

**Authors:** Gaoyang Zhu, Junlin Feng, Yunjun Zhang, Xinhua Sun, Shucheng Liang, Bin Wang, Muzhi Gao

**Affiliations:** 1 College of Electronic and Information Engineering, Shandong University of Science and Technology, Qingdao 266590, China; gaoyangzhu@sdust.edu.cn (G.Z.); 202483130080@sdust.edu.cn (J.F.); 202483130144@sdust.edu.cn (Y.Z.); 202583130103@sdust.edu.cn (X.S.); 202582130032@sdust.edu.cn (S.L.); 2 College of Control Science and Engineering, China University of Petroleum (East China), Qingdao 266580, China; wangbin2015@upc.edu.cn

**Keywords:** broadband microwave sensing, ridged horn antenna, water-cut measurement, oil-water emulsion, stratified flow

## Abstract

**Highlights:**

**What is the main finding?**

- The present study improves the frequency limitation of conventional horn antennas in pipeline water-cut detection, in which ridge structures lowers the cutoff frequency of the horn antenna and achieves broadband impedance matching, enabling water-cut measurement over a broader frequency range.

**What are the implications of the main findings?**

- This work proposes a non-contact water-cut monitoring system that supports a broader frequency band.
- The frequency range of water-cut detection in this system covers a broader dielectric dispersion spectrum of liquid water.

**Abstract:**

As oil and gas reservoirs progress into the mid-to-late stages of development, produced fluids increasingly exhibit high water-cut and complex flow regimes. Conventional water-cut measurement techniques based on capacitance, conductance, and resistance often face challenges in terms of accuracy, stability, and adaptability. In this study, a novel non-contact broadband microwave system, based on a ridged-horn antenna microwave transmission sensor (RHAMTS), is proposed to achieve highly sensitive full-range (0–100%) water-cut monitoring. The RHAMTS consists of two identical ridged-horn antennas, whose geometries are optimized through analytical design calculations and full-wave finite-element simulations. Numerical simulations are first performed to elucidate the sensing mechanism. Subsequently, static and dynamic experiments are conducted under two representative conditions: emulsified oil-water mixtures and stratified oil-water layers. The results indicate that the broadband spectral signatures of the RHAMTS can effectively characterize water-cut in both emulsified mixtures and stratified oil-water layers. For emulsified mixtures, both amplitude attenuation and phase shift vary systematically with water-cut, and the RHAMTS can still effectively characterize water-cut under saline conditions. For stratified oil-water flow, results from both static and dynamic experiments demonstrate that amplitude attenuation provides more robust features for practical water-cut discrimination. Compared with conventional methods, the proposed RHAMTS offers non-contact operation, rich spectral information, and compatibility with various flow regimes, providing a feasible and efficient approach for water-cut monitoring under complex field conditions.

## 1. Introduction

As oil and gas reservoirs advance into the mid-to-late stages of development, produced fluids generally exhibit increasing water-cut and more complex flow regimes [1]. Under such conditions, oil-water two-phase flow and even oil-water-gas multiphase flow become commonplace, and the produced fluids exhibit complex compositions and dynamics [2,3]. Accurately determining the oil-water ratio in real time is therefore essential for refined injection-production management, reservoir surveillance, and enhanced recovery.

At present, capacitance-, conductance-, and resistance-based methods remain the most widely used techniques for water-cut measurement [4,5,6,7,8]. However, because field conditions often involve complex and evolving flow regimes and high water-cut, these conventional sensors suffer from several limitations: (i) measurement accuracy and calibration stability degrade as the flow pattern and phase distribution change; (ii) the applicable range across different flow regimes is narrow; and (iii) electrodes and contact probes are prone to polarization, fouling, and corrosion, which complicates long-term deployment both downhole and at the surface [9,10,11,12,13]. These issues motivate the development of non-contact sensing technologies with improved adaptability to varying multiphase-flow conditions [14].

Microwave techniques have attracted increasing attention for water-cut and multiphase-flow measurements because they are sensitive to the medium’s complex permittivity and can operate in non-contact settings [15]. A variety of microwave sensor structures have been reported, including cavity perturbation sensors, planar resonant sensors based on microstrip/patch antennas, and transmission-line sensors [16,17,18]. For example, Wei et al. utilized phase shift in a conical helical transmission line, and a single sensor operating at 80 MHz achieved full-range (0–100%) measurement with a reported resolution better than 3% [19]. Sharma et al. proposed a cylindrical cavity resonator to estimate the dielectric constant of oil-water mixtures with concentrations ranging from 0 to 5% by tracking the resonance shifts of the TM_010_ and TM_011_ modes [20]. Li et al. reported a microwave transmission method for oil-water two-phase mixtures, achieving detection over a 30–75% water content by analyzing transmission magnitude and phase [21]. Chen et al. developed a semi-closed cylindrical resonator operating in a narrowband (9.302–9.307 GHz) for low-water-content detection in oil [22]. Zeng et al. employed a horn antenna-based transmission method in the 8–12 GHz band for low-water-content measurement below 1% [23].

Despite these advances, many microwave-based approaches remain narrowband or single-frequency designs and may provide reliable performance only within limited ranges or under relatively stable phase distributions. Their robustness may degrade when the flow regime changes or when the phase distribution becomes strongly non-uniform [24,25]. In contrast, broadband measurements can provide richer spectral information by sweeping a wide frequency range and observing frequency-dependent transmission signatures. Different frequencies may exhibit different sensitivities to effective permittivity, interface distribution, and flow regime, thereby offering the possibility of identifying representative frequency points suitable for practical calibration and water-cut estimation [26]. For instance, Xu et al. designed a broadband antenna configuration over 0.1–6 GHz and showed that both S_21_ magnitude and phase vary with water content; however, their design requires direct contact with the fluid, which increases the risk of blockage and contamination during long-term operation [27]. These limitations reduced the practicality of the existing method for reliable water-cut monitoring under realistic production conditions.

Motivated by these considerations, this study proposes a new ridged-horn antenna-based microwave transmission sensor (RHAMTS), for non-contact, broadband, full-range (0–100%) water-cut monitoring in oil-water pipelines. In addition to non-contact operation, the system provides rich broadband spectral information, which is beneficial for feature selection and subsequent water-cut estimation. Compared with other antenna types, the ridged-horn antenna is particularly suitable for the present application because it offers broad impedance bandwidth, directive radiation, and convenient external installation in a face-to-face transmission configuration. These characteristics are advantageous for maintaining stable alignment and repeatable measurements in practical pipeline environments. To validate the proposed method, simulations and experiments are carried out under two representative conditions: (i) emulsified oil-water mixtures with water-cut ranging from 0% to 100%, which can be approximated as homogeneous effective media, and (ii) stratified oil-water layers across the same full range. For the stratified case, both vertical and horizontal antenna orientations relative to the interface are examined to evaluate the impact of coupling conditions on measurement regularity. In addition to static measurements, dynamic experiments under stratified oil-water flow are further conducted to assess the robustness of the RHAMTS under flowing conditions and to support its practical relevance for pipeline monitoring.

The results show that emulsified mixtures exhibit systematic broadband signatures in both amplitude attenuation and phase shift, enabling clear water-cut dependence across the frequency band. The RHAMTS also remains responsive under saline conditions, indicating its applicability beyond pure oil-water mixtures. For stratified oil-water flow, results from both static and dynamic experiments demonstrate that amplitude attenuation provides more robust features for practical water-cut discrimination. The influence of antenna orientation is also clarified, which helps guide feature selection and sensor deployment under layered flow conditions. Overall, the results indicate that the proposed RHAMTS can reliably capture spectral features associated with water-cut under various oil-water distributions and flow conditions, providing a feasible basis for practical water-cut monitoring in complex field environments.

## 2. Theory of the System

### 2.1. Dielectric Properties of Oil-Water Mixtures

Oil-water systems encountered in production pipelines consist of two electrically distinct phases: a low-permittivity, weakly polar oil phase and a high-permittivity, strongly polar water phase. The dielectric properties of oil and gas are relatively close to each other compared with those of water, and therefore, oil and gas can be treated as an equivalent low-permittivity phase. At microwave frequencies, typical crude oils exhibit a dielectric constant in the range of 2–3, whereas water has a much higher dielectric constant of approximately 80 at room temperature. This pronounced dielectric contrast constitutes the fundamental physical basis for microwave-based water-cut measurement.

Water is a polar liquid whose permittivity exhibits pronounced frequency dispersion, which can be described by the Debye relaxation model [28]. The complex permittivity of water is expressed as

(1) $$\varepsilon \left(\omega \right)=\varepsilon_{\infty }+\left(\varepsilon_{s}-\varepsilon_{\infty }\right)/\left(1+j\omega \tau \right)$$

where $\varepsilon_{s}$ and $\varepsilon_{\infty }$ denote the static and high-frequency permittivity limits, and *τ* denotes the molecular relaxation time. In contrast, crude oil is mainly composed of non-polar hydrocarbons, resulting in weak polarization, low permittivity, and low dielectric loss.

For an oil-water mixture, the effective complex permittivity depends primarily on the volumetric fraction of water. When the dispersed droplets are sufficiently small or uniformly dispersed-as in emulsified mixtures-the mixture can be approximated using classical effective-medium theories [29]. A commonly adopted mixing model is expressed as

(2) $$\sqrt{\varepsilon_{eff}}=q\sqrt{\varepsilon_{w}}+(1-q)\sqrt{\varepsilon_{o}}$$

where $\varepsilon_{w}$ and $\varepsilon_{o}$ represent the relative permittivity of pure water and pure oil, respectively, and ***q*** denotes the volumetric water fraction.

As the water fraction increases, the proportion of polar molecules in the mixture rises, leading to enhanced polarization, higher effective permittivity, and increased dielectric losses. These variations directly influence the propagation characteristics of microwave signals, including both amplitude attenuation and phase shift.

**Figure 1:**
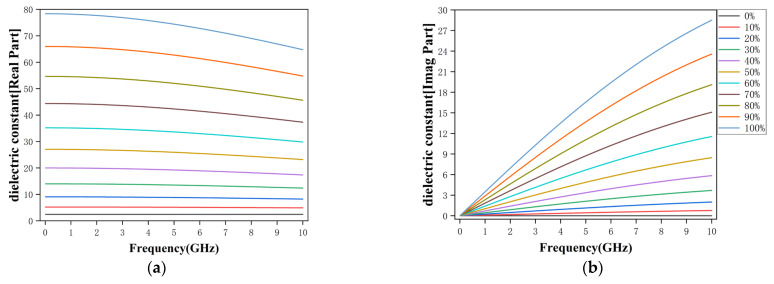
Calculated effective relative permittivity. (**a**) Real part. (**b**) Imaginary part.

By substituting the permittivities of pure oil and water into the effective permittivity model, the frequency-dependent effective permittivity and attenuation behavior of oil-water mixtures with different water-cuts can be evaluated [30]. Figure 1 illustrates the calculated effective relative permittivity and the corresponding attenuation factor for water-cuts ranging from 0% to 100%. As the water fraction increases, the effective permittivity increases markedly, especially at medium and high water-cuts, while its dispersion over the 2–8 GHz range remains relatively weak except for nearly pure water. In contrast, the attenuation factor increases almost linearly with frequency and is strongly dependent on water-cut. These results indicate that increasing water content simultaneously increases $\varepsilon_{eff}^{\prime }$ and $\varepsilon_{eff}^{\prime \prime }$, thereby reducing the propagation velocity and enhancing the attenuation of the transmitted microwave signal. Consequently, both the amplitude attenuation and phase shift of the transmitted signal exhibit monotonic variations with water-cut at appropriately selected frequencies, forming the physical basis for microwave transmission-based water-cut sensing.

### 2.2. Sensing Principle

**Figure 2:**
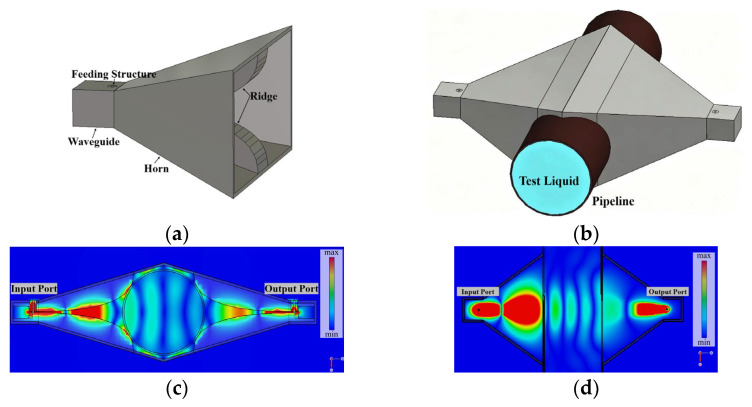
(**a**) Configuration of the proposed broadband ridged-horn antenna. (**b**) Configuration of the broadband RHAMTS setup for water-cut monitoring in a pipeline. (**c**) Electric field distributions on the E-plane for the proposed RHAMTS system. (**d**) Electric field distributions on the H-plane for the proposed RHAMTS system.

In the proposed RHAMTS configuration, a pair of broadband ridged-horn antennas is positioned on opposite sides of a 50 mm non-metallic pipe, as illustrated in Figure 2a. One horn antenna operates as the transmitter, radiating a directive microwave beam toward the pipe, while the other antenna receives the transmitted field after it propagates through the pipe wall and the oil-water mixture. The fluid-filled pipe section between the two antennas, therefore, forms a short effective transmission channel, whose electromagnetic properties are governed by the effective complex permittivity of the fluid under test.

Figure 2c,d show the electric field distributions of the antenna and pipe in the E-plane and H-plane, respectively, thus providing a complete representation of the three-dimensional propagation characteristics. The results indicate that, for the selected geometry, the majority of the transmitted energy is confined within the dominant transmission region that traverses the pipe aperture and couples efficiently into the receiving antenna, thereby allowing a more comprehensive understanding of microwave propagation through the oil-water mixture. As the water-cut increases, both the effective permittivity and dielectric loss of the oil-water mixture increase, leading to stronger field attenuation within this transmission region and a larger accumulated phase delay between the two antennas. These effects are directly reflected in the measured complex transmission coefficient S_21_.

The propagation of the microwave signal along the effective path can be described by an effective complex propagation constant *γ_eff_*,

(3) $$\gamma_{eff}=\alpha_{eff}+j\beta_{eff}$$

where *α_eff_* is the effective attenuation constant, and *β_eff_* is the effective phase constant. For a weakly lossy dielectric channel, both *α_eff_* and *β_eff_* increase monotonically with $\varepsilon_{eff}^{\prime }$ and $\varepsilon_{eff}^{\prime \prime }$.

At a given frequency, a larger *α_eff_* results in stronger amplitude attenuation, while a larger *β_eff_* produces a greater phase shift [31,32]. Increasing water content leads to higher effective permittivity and dielectric loss, thereby enhancing both amplitude attenuation and phase shift along the propagation path. These characteristics constitute the theoretical foundation of water-cut sensing using the RHAMTS.

## 3. System Configuration

### 3.1. Sensor Design and Modeling

**Figure 3:**
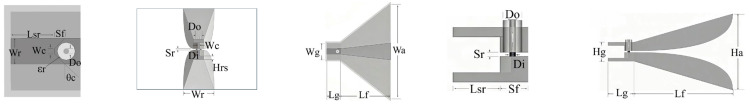
Detailed structure of the proposed wideband ridged-horn antenna.

The proposed sensing system employs a pair of broadband ridged-horn antennas as the transmitting and receiving elements. The antenna configuration is shown in Figure 2a, with the detailed geometry and key design parameters summarized in Figure 3. The antenna consists of a rectangular waveguide feed, a pair of symmetrically tapered ridges, and a flared horn section. The ridges extend from the waveguide throat into the horn region and gradually taper toward the aperture, enhancing the impedance bandwidth to match the free-space impedance while maintaining a compact overall size [33,34].

This configuration ensures broad frequency coverage with minimal size constraints. In Figure 3, *Wg* and *Hg* denote the width and height of the waveguide, while *Lg* represents the length of the ridge-waveguide transition, and *Lf* is the flare length. The design dimensions of the proposed ridged-horn antenna are summarized in Table 1. The antenna is fed by two coaxial feedlines, each designed to have a characteristic impedance of 50 Ω. The flare profiles of both the outer plates and the ridges are described by smooth curves, which help reduce reflections and achieve a gradual transition from the waveguide mode to free-space radiation [35]. All metallic parts are modeled as perfect electric conductors in the simulation and subsequently fabricated from aluminum alloy sheets in the prototype.

### 3.2. Antenna Performance

**Figure 4:**
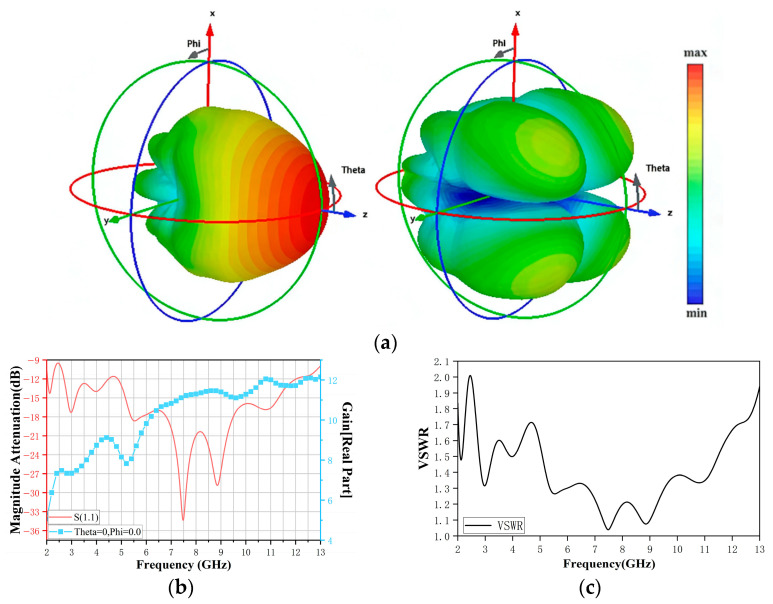
(**a**) 3-D gain pattern and cross-polarization of the antenna obtained by simulation. (**b**) S_11_ response curve and (**c**) VSWR of the ridged-horn antenna.

The radiating characteristics of the ridged horn are analyzed using full-wave electromagnetic simulation. Figure 4a shows the 3-D gain pattern and the cross-polarization distribution of the antenna. The radiation pattern exhibits a single dominant lobe along the main beam direction, with relatively low sidelobe levels, ensuring efficient coupling through the pipe cross-section. The cross-polarization level remains low, indicating that the radiated field is predominantly co-polarized and aligned with the E-plane (ridge) direction of the horn antenna. These results indicate that the cross-polarized components have a negligible effect on the accuracy of water-cut measurement.

Figure 4b,c show the simulated input reflection coefficient S_11_, along with the realized main lobe gain and voltage standing-wave ratio (VSWR) as functions of frequency. The reflection coefficient remains below approximately −10 dB over most of the 2–12 GHz band, indicating excellent broadband impedance matching between the horn and the 50 Ω feed. The realized gain remains moderate across the band, providing sufficient link budget while avoiding an excessively narrow beam. These results confirm that the designed ridged-horn antenna satisfies the bandwidth and radiation requirements of the proposed sensing application.

### 3.3. Design and Simulation of Measurement System

For water-cut sensing, two identical ridged horns are placed face-to-face at symmetrically opposite positions on both sides of a 50 mm non-metallic pipe, and are fed through waveguide ports. The centers of the horn apertures are located 19 mm from the pipe center. The non-metallic pipe has a wall thickness of 2 mm and a length of 200 mm, and is in contact with the ridges, as illustrated in Figure 2b. The pipe is filled with oil-water mixtures, and the horns are coaxially aligned with the pipe to maximize the overlap between the main beam and the pipe aperture. In the simulations, the antenna material is modeled as an ideal conductor, while the feeding dielectric and the pipe are assigned as insulating materials of polytetrafluoroethylene (PTFE) and polymethyl methacrylate (PMMA), respectively. Different effective permittivities corresponding to various water contents are assigned to the fluid region using the effective permittivity model. After propagation through the mixture, the microwave signal transmitted by the transmitting antenna is received by the receiving antenna. This setup enables measurement of the amplitude attenuation and phase shift of the electromagnetic wave propagating through the mixture. A frequency sweep from 2 to 12 GHz is applied in the simulations.

**Figure 5:**
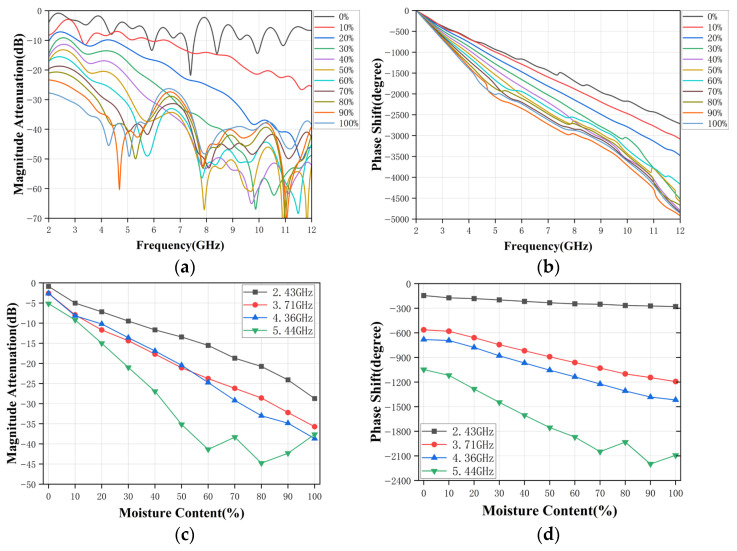
Simulated response of the RHAMTS system. (**a**) Broadband amplitude attenuation. (**b**) Broadband phase shift. (**c**) Single-frequency amplitude attenuation. (**d**) Single-frequency phase shift.

Simulated results are summarized in Figure 5, showing the amplitude attenuation and phase shift versus frequency for water-cuts ranging from 0% to 100%. As the water fraction increases, the overall transmission level decreases due to stronger dielectric loss. The phase decreases monotonically with frequency and becomes more negative with increasing water-cut, consistent with the mixture’s higher effective permittivity and slower propagation velocity. In the 2–8 GHz region, the amplitude attenuation curves for different water-cuts are well separated yet smooth, while the unwrapped phase decreases almost linearly with frequency for each water-cut level. In this band, the ordering of the curves with respect to water-cut is preserved, and the response is minimally affected by slight variations in geometry or alignment, which is advantageous for robust calibration.

However, at higher frequencies (above approximately 8 GHz), the simulated responses become noticeably more irregular. Additional resonant notches and rapid fluctuations appear in the amplitude attenuation, and the phase shift curves start to exhibit local distortions and partial crossings between different water-cut cases. This behavior is attributed to stronger multiple reflections at the pipe interfaces, increased sensitivity to small geometrical tolerances, and the reduced electrical size of the horn aperture relative to the wavelength at the lower end of the band. Consequently, the 2–8 GHz band is chosen for the subsequent experimental study.

To further evaluate the sensing capability, several representative frequencies (e.g., 2.43, 3.71, 4.36, and 5.44 GHz) are selected from the broadband response, and the transmission amplitude attenuation and the phase shift at these frequencies are plotted as functions of water-cut in Figure 5c and Figure 5d, respectively. The results show that for emulsified mixtures, both amplitude attenuation and phase shift exhibit clear, monotonic trends with increasing water content. At certain frequencies, these relationships are nearly linear over most of the 0–100% range, which is favorable for subsequent calibration and model fitting.

These simulation results demonstrate that the proposed RHAMTS, when configured as a transmitting-receiving pair across the pipe, provides a stable broadband link with an S_21_ response that is highly sensitive to the dielectric properties of the oil-water mixture. This configuration underpins the experimental validation and data analysis presented in the following section.

## 4. Experiment Validation

### 4.1. Fabrication of the Ridged-Horn Antennas

**Figure 6:**
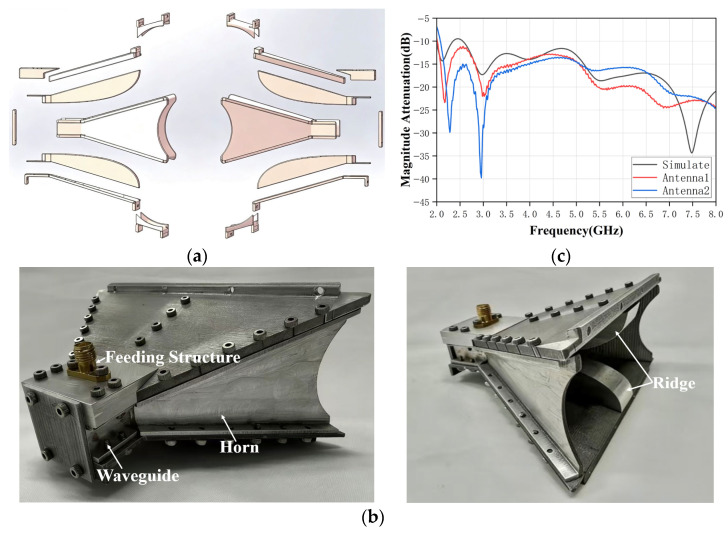
(**a**) Decomposed multi-layer structure of the ridged-horn antenna. (**b**) Assembled prototype. (**c**) Comparison of S_11_ between simulated and fabricated antennas.

To experimentally validate the proposed sensing approach, a pair of ridged-horn antennas is fabricated according to the design parameters. The antenna structure is decomposed into multiple planar metal plates, which are assembled to form the ridged-horn antenna, as shown in Figure 6a. Photographs of the assembled antennas are presented in Figure 6b, demonstrating that the fabricated prototypes closely match the designed geometry and exhibit robust structural integrity.

The reflection coefficient of the fabricated antenna is measured using a vector network analyzer (VNA, T5280A, TRANSCOM, Shanghai, China) and compared with the results of full-wave simulations. As shown in Figure 6c, the measured S_11_ curves for both prototypes closely follow the simulated trend across the 2–8 GHz frequency range. Minor discrepancies between the measured and simulated results can be attributed to fabrication tolerances, such as slight misalignments during assembly, small gaps between components, and surface roughness. Despite these factors, both prototypes maintain an S_11_ value below −10 dB for most of the operating band, confirming that the antennas retain good broadband impedance matching and are suitable for transmission experiments.

### 4.2. Experimental Setup

The experimental procedure is as follows: First, the VNA is powered on and allowed to warm up for 15 min. A standard two-port SOLT calibration is then performed, in which each port undergoes Open, Short, and Load measurements sequentially, followed by a Thru measurement between the two ports, in order to minimize system noise and measurement errors. Once the calibration is complete and the antennas are securely mounted, the two antennas are connected to the VNA via SMA interfaces, and the control computer is connected to ensure proper data acquisition. Subsequently, oil-water samples of the desired proportions are prepared and injected into the non-metallic pipe positioned between the two antennas. Finally, the measurement data are recorded.

**Figure 7:**
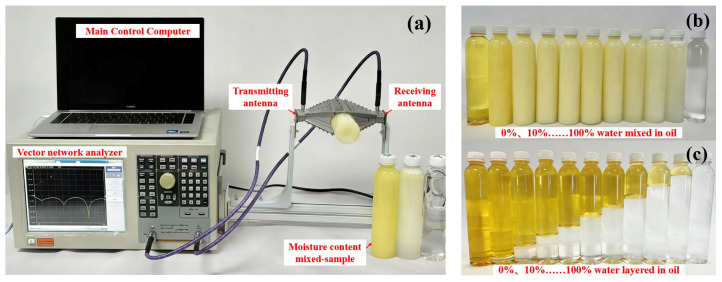
(**a**) General photograph of the experimental installations for RHAMTS measurement. (**b**) Samples of oil-water emulsion. (**c**) Samples of stratified oil-water.

The complete RHAMTS measurement system is shown in Figure 7a. The two ridged-horn antennas are placed face to face and mounted on a rigid aluminum frame to ensure precise alignment during measurements. A non-metallic pipe is placed between the antennas and filled with oil-water mixtures with a known water-cut. The VNA is used to record the complex transmission coefficient S_21_ over the 2–8 GHz frequency band. Each measurement is repeated three times, and the mean is used to ensure repeatability and reduce random variation.

**Figure 8:**
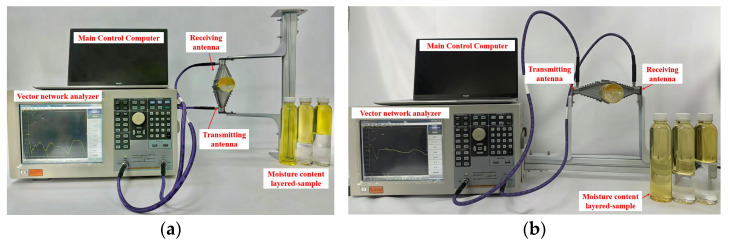
Experimental setup for oil-water stratified flow. (**a**) Vertical antenna placement. (**b**) Horizontal antenna placement.

**Figure 9:**
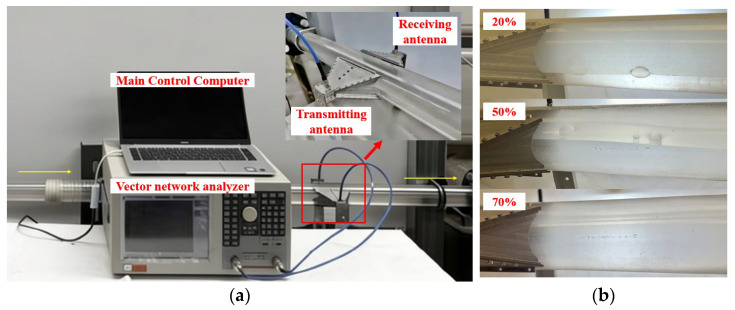
(**a**) Experimental setup for dynamic oil-water stratified pipe flow. (**b**) The pipeline contains stratified oil and water layers.

(1) *Oil-Water Emulsions*. For the emulsified oil-water mixtures, pure water and vegetable oil are blended using Tween 80(Macklin, Shanghai, China) as an emulsifier. The mixture is thoroughly stirred to create a homogeneous emulsion with minimal spatial variation, ensuring that the dielectric properties are consistent during measurement. The experimental setup for emulsions is shown in Figure 7a, along with photographs of the prepared emulsions at different water-cut levels.

(2) *Saline Oil-Water Emulsions*. For the homogeneous emulsion prepared by stirring with Tween 80 as the emulsifier, a fixed amount of sodium chloride is added to achieve a salinity of 2000 mg/L. The mixture is thoroughly stirred to ensure complete dissolution of the salt. The experimental setup is shown in Figure 7a, and this configuration is used to evaluate the response of the RHAMTS to water-cut under saline conditions.

(3) *Oil-Water Stratified Flow*: *Vertical Antenna Placement*. In the vertical antenna placement configuration, as depicted in Figure 8a, the horn antennas are oriented perpendicular to the flow direction, with the microwave signal first propagating through the water phase and then through the oil phase. This setup investigates the effects of the different flow phases and the signal’s propagation characteristics as it traverses the two distinct media.

(4) *Oil-Water Stratified Flow*: *Horizontal Antenna Placement*. The experiment setup for horizontal oil-water two-phase flow is shown in Figure 8b. In this configuration, the two ridged-horn antennas are placed parallel to each other, with the electromagnetic waves propagating horizontally through the oil-water mixture. This setup enables the examination of how the microwave signal is affected by stratified flow and the interaction between the oil and water phases.

(5) *Dynamic Experiment*: *Oil-Water Stratified Flow with Horizontal Antenna Placement.* The experimental setup for horizontal oil-water two-phase flow is shown in Figure 9a. In this dynamic pipeline configuration, samples with different oil-water fractions are prepared using 3# industrial white oil and purified water and pumped into the pipeline by liquid pumps. Stratification between oil and water can be achieved at a flow velocity below 0.1 m/s inside the pipeline. The pipeline is made of low-loss PMMA, with an inner diameter of 55 mm and a wall thickness of 4 mm. Two ridged-horn antennas are placed parallel to each other on the horizontal section of the pipe, allowing electromagnetic waves to propagate horizontally through the oil-water mixture. This setup enables the investigation of how microwave signals are affected by stratified flow and the interactions between the oil and water phases.

### 4.3. Results and Discussion

In this section, the experimental results for emulsified oil-water mixtures and stratified oil-water flow are analyzed. The broadband response in the 2–8 GHz band and the frequency-dependent signatures of the measured S_21_ are used to validate the RHAMTS and assess the feasibility of broadband microwave transmission for water-cut measurement under different flow conditions.

**Figure 10:**
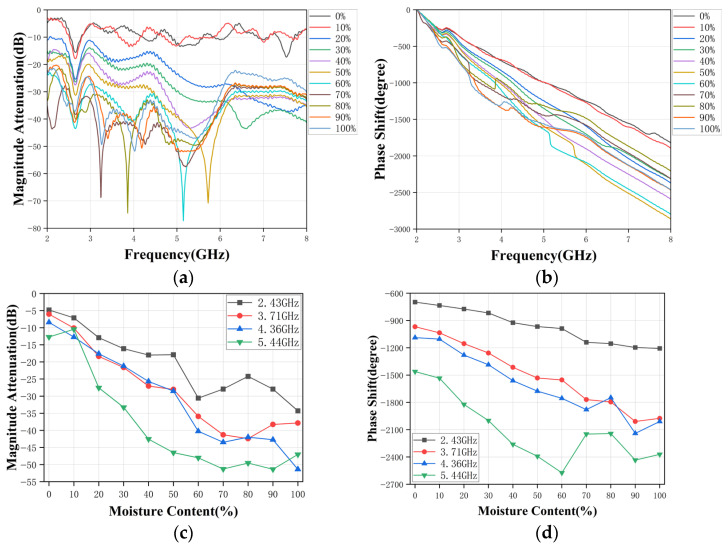
Measured response of RHAMTS for oil-water emulsions. (**a**) Broadband amplitude attenuation. (**b**) Broadband phase shift. (**c**) Single-frequency amplitude attenuation. (**d**) Single-frequency phase shift.

(1) *Oil-Water Emulsions*. Figure 10a,b show the measured broadband amplitude attenuation and phase shift for emulsions with water content ranging from 0% to 100%. As predicted by the simulation trends in Section 3, the overall attenuation of S_21_ decreases systematically across most of the band as water content increases, consistent with higher effective permittivity and stronger dielectric loss in the mixture. Although spectral ripples and localized notches are visible-particularly in the mid-band-distinct “frequency-dependent fingerprints” can still be observed for different water-cuts, implying that the broadband response contains sufficient information for feature extraction.

The curves for different water-cuts remain clearly separated over a wide frequency range, confirming that the broadband response is strongly sensitive to the dielectric properties of the emulsion. The corresponding phase shift responses in Figure 10b decrease approximately linearly with frequency for each water-cut level. Mixtures with higher water content exhibit more negative phase delays, in agreement with the theoretical discussion in Section 2. This trend reflects the increased effective permittivity and reduced phase velocity in mixtures with higher water content, resulting in a larger accumulated phase delay between the two antennas.

To evaluate the suitability of specific frequencies for practical water-cut estimation, the amplitude attenuation and phase shift at four representative frequencies (2.43, 3.71, 4.36, and 5.44 GHz) are extracted from the broadband response and plotted as functions of water-cut, as shown in Figure 10c,d. Both amplitude attenuation and phase shift exhibit clear monotonic trends over the whole 0–100% range. At 2.43, 3.71, and 4.36 GHz, the relationships are nearly linear, which makes them suitable for subsequent calibration or for implementing simple regression-based inversion. At 5.44 GHz, increased variability is observed due to higher sensitivity to alignment and mixture homogeneity, although the different water-cut levels remain well separated. These results demonstrate that the broadband measurement not only provides rich spectral information, but also allows the identification of some “good” frequencies suitable for single- or multi-frequency feature-based estimation.

**Figure 11:**
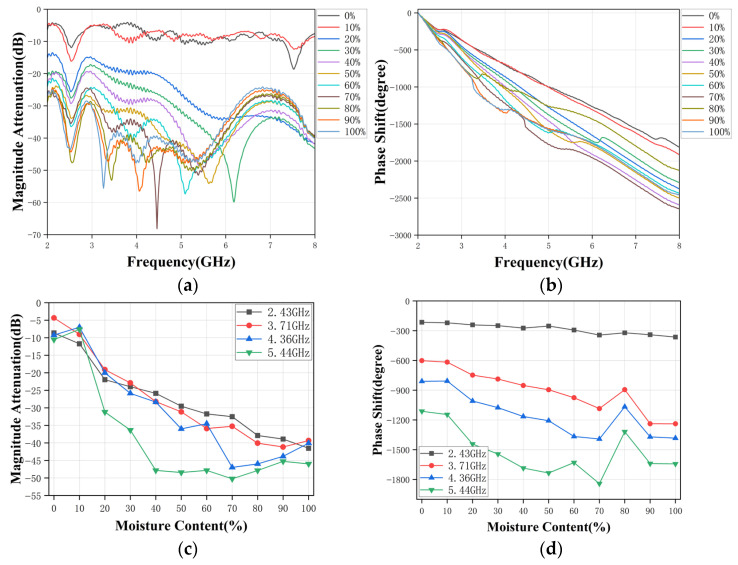
Measured response of RHAMTS for saline oil-water emulsions. (**a**) Broadband amplitude attenuation. (**b**) Broadband phase shift. (**c**) Single-frequency amplitude attenuation. (**d**) Single-frequency phase shift.

(2) *Saline Oil-Water Emulsions.* Figure 11a,b present the measured broadband amplitude attenuation and phase shift of saline oil-water emulsion samples with water contents ranging from 0% to 100% at a salinity of 2000 mg/L. As the water-cut increases, the amplitude attenuation of S_21_ decreases systematically across most of the frequency band, while the corresponding phase shift exhibits a clear and regular variation with frequency. Similar to the non-saline emulsions, the saline emulsions also exhibit distinguishable broadband spectral responses for different water-cut levels, indicating that the RHAMTS remains sensitive to water-cut even in the presence of dissolved salt.

To further examine the effect of salinity, Figure 11c,d compare the single-frequency responses of saline and non-saline emulsions. Compared with pure-water emulsions, the saline emulsions exhibit slightly increased amplitude attenuation and marginally larger phase shifts. This difference can be attributed to the higher conductivity and dielectric loss introduced by the dissolved sodium chloride. Nevertheless, the monotonic dependence of both amplitude attenuation and phase shift on water-cut remains well preserved. These results indicate that the addition of salinity mainly changes the absolute transmission response, whereas the qualitative dependence of the measured response on water-cut remains unchanged. This suggests that moderate variations in water conductivity, such as those introduced by saline water, do not compromise the ability of the RHAMTS to characterize water-cut. Therefore, the RHAMTS can still effectively characterize water-cut under saline conditions.

**Figure 12:**
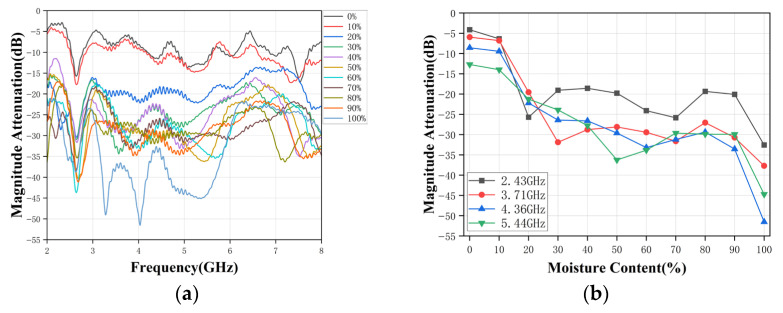
(**a**) Broadband response with vertically placed antennas. (**b**) Single-frequency response with vertically placed antennas.

(3) *Oil-Water Stratified Flow*: *Vertical Antenna Placement*. Figure 12a shows the measured broadband amplitude attenuation for stratified oil-water layers with vertically placed antennas. Compared with emulsified mixtures, stratified flow constitutes a strongly non-homogeneous medium in which the interface location and the layered geometry influence the electromagnetic field distribution and the effective propagation path.

As a result, the broadband amplitude attenuation spectra exhibit pronounced ripples and localized deep notches, and the ordering of amplitude attenuation curves with respect to water-cut is partially degraded in several sub-bands. The single-frequency amplitude attenuation at selected frequencies is extracted and plotted versus water-cut in Figure 12b. The results show an overall decreasing tendency, but also exhibit local non-monotonicity and plateau behaviors across the medium-to-high water-cut range. This indicates that the measured attenuation is determined not only by bulk dielectric loss but also by frequency-dependent coupling conditions and residual multipath interference associated with reflections at the pipe boundaries and the oil-water interface. The irregularity becomes more evident at high water-cuts, where the transmission channel is dominated by the high-loss water layer and the sensitivity to small geometric perturbations is amplified.

**Figure 13:**
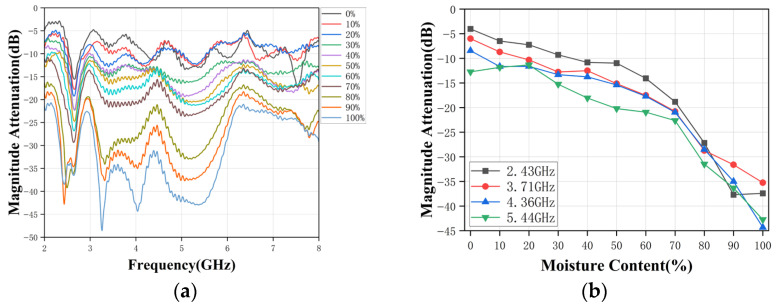
(**a**) Broadband response with horizontally placed antennas. (**b**) Single-frequency response with horizontally placed antennas.

(4) *Oil-Water Stratified Flow*: *Horizontal Antenna Placement*. Figure 13a,b summarize the results for stratified oil-water flow when the antennas are placed horizontally on opposite sides of the pipe. Compared with the vertical case, the broadband amplitude attenuation response in Figure 13a shows improved separation and more consistent ordering over a larger portion of the 2–8 GHz band. Although spectral ripples and notches are still present-as expected for a practical finite-length transmission channel-the overall attenuation level decreases more systematically with increasing water-cut. This indicates that the horizontal placement yields a more stable mapping between water-cut and amplitude attenuation, which is advantageous for measurement.

As shown in Figure 13b, the amplitude attenuation of S_21_ at 2.43, 3.71, 4.36, and 5.44 GHz exhibits a clear monotonic decrease over the entire 0–100% range, with a relatively gradual decrease at low-to-medium water-cuts and a pronounced attenuation drop beyond approximately 70%. This monotonicity is highly favorable for calibration and inversion using simple monotonic regression or lookup-table methods. Among the selected points, higher-frequency features exhibit greater attenuation contrast at higher water-cuts (i.e., greater sensitivity). In contrast, the lower-frequency feature (2.43 GHz) is less affected by notch-induced perturbations and may offer improved robustness.

Taken together, these observations indicate that horizontal antenna placement provides more stable and predictable transmission characteristics in stratified flows. Consequently, the horizontal configuration is recommended for future implementations of the proposed sensing system, particularly in high-water-cut and strongly stratified flow regimes.

**Figure 14:**
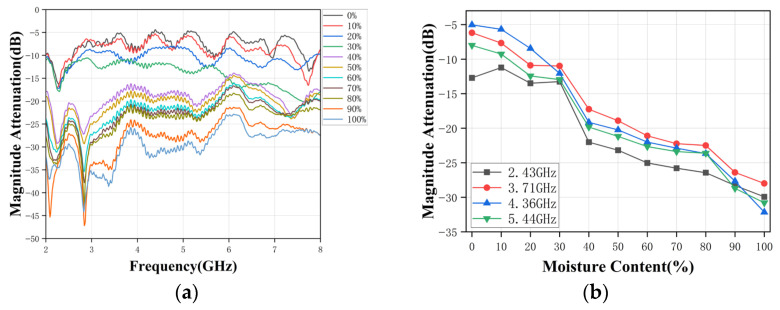
Dynamic experiment: oil-water stratified flow. (**a**) Broadband response with horizontally placed antennas. (**b**) Single-frequency response with horizontally placed antennas.

(5) *Dynamic Experiment*: *Oil-Water Stratified Flow with Horizontal Antenna Placement.* The measured broadband and single-frequency responses for horizontal stratified oil-water flow are presented in Figure 14a,b. Similar to the static experiments in Section 4, the broadband response exhibits clear separation among different water-cuts, and the single-frequency attenuation trends remain monotonic across the full 0–100% range. Compared with the static case, the high-water-cut region shows slightly lower attenuation in the dynamic flow experiment, with a maximum difference of approximately 10 dB at 100% water content. The amplitude difference observed at high water-cut can be attributed to two contributing factors. The first is the difference in the oils used in the two experiments: the vegetable oil employed in the static experiment exhibited higher dielectric loss than the white oil used in the dynamic experiment. The second is the difference in the dimensions of the non-metallic pipes: the static experiment utilized a pipe with an inner diameter of 50 mm and a wall thickness of 2 mm, whereas the dynamic experiment used a pipe with an inner diameter of 55 mm and a wall thickness of 4 mm. Considering the horn antenna height of 49 mm, a smaller pipe diameter results in a larger gap between the horn and the pipe edge, which enhances electromagnetic-wave scattering. In contrast, the larger pipe diameter in the dynamic experiment better covers the main lobe of the antenna beam, thereby improving coupling efficiency and resulting in lower attenuation compared with the static experiment.

Despite these variations, the overall trends remain systematic and repeatable, demonstrating that the RHAMTS system can still provide reliable water-cut estimation under flowing conditions. Horizontal antenna placement provides a more stable and predictable mapping between water-cut and amplitude attenuation, confirming its suitability for practical pipeline monitoring in dynamic, stratified two-phase flows.

### 4.4. Experimental Analysis

In the RHAMTS microwave transmission method, to verify the stability of the response for oil-water samples across the full water-cut range (0–100%), the error in the amplitude response of the transmission coefficient S_21_ is analyzed under both static and dynamic experimental conditions, as shown in Table 2. Based on the results of Experiments (1) to (5), the microwave amplitude attenuation becomes more pronounced, and the phase delay increases with increasing water content. In this study, a quadratic polynomial model relating amplitude to water-cut was established for emulsified oil-water systems, enabling the estimation of water content using the following equation:

(4) $$y=a_{1}+a_{2}x+a_{3}x^{2}$$

where *y* denotes the water-cut value, and *x* denotes the transmission coefficient amplitude measured in different experiments.

The R^2^ values of the fitted models indicate that Experiments (1), (2), (4), and (5) achieved high fitting accuracy (*R^2^* > 0.96), demonstrating that the proposed polynomial models can reliably capture the relationship between amplitude and water-cut under most experimental conditions. To further assess the reproducibility of the measurements, the performance of the water-cut prediction model was quantitatively evaluated by calculating the relative error as ∣(*x_rep_* − *x*_pre_)/*x*_pre_∣ × 100%, where *x_rep_* represents the amplitude value from repeated experiments, and *x_pre_* denotes the value predicted by the model. This analysis highlights the error associated with repeated measurements, confirming that the model provides consistent and reliable water-cut predictions despite experimental variability.

**Figure 15:**
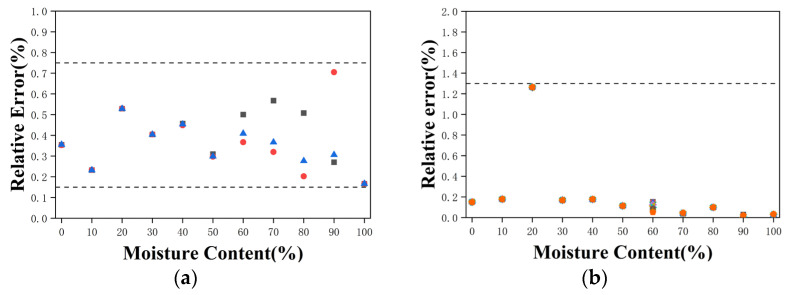
Relative error between repeated experiments and the fitted model. (**a**) Oil-water emulsions. (**b**) Saline oil-water emulsions.

Figure 15 shows the relative errors obtained from the water content prediction models for the oil-water emulsion and saline oil-water emulsion experiments. For the oil-water emulsion experiment, data points have relative errors ranging from 0.16% to 0.71%, while for the saline oil-water emulsion experiment, data points have relative errors ranging from 0.05% to 1.32%.

**Figure 16:**
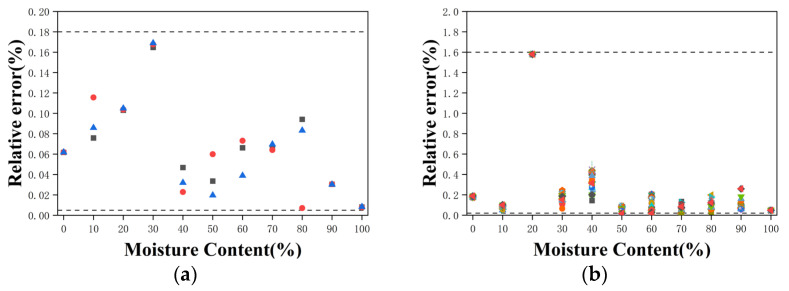
Relative error between repeated experiments and the fitted model. (**a**) Oil-water stratified flow-horizontal antenna placement. (**b**) Dynamic experiment-oil-water stratified flow-horizontal antenna placement.

The relative errors between repeated oil-water stratified experiments and the fitted model predictions are shown in Figure 16. For the static measurements, data points exhibit relative errors ranging from 0.05% to 0.17%, while for the dynamic measurements, data points fall within 0.55% to 1.59%. These results indicate that the relative errors are small, that the predicted data from static and dynamic stratified experiments are in good agreement, and that the experimental procedure demonstrates excellent repeatability.

Additionally, to demonstrate the practicality and functionality of the RHAMTS, the relevant performance characteristics and measurement methods of different sensors are summarized. Table 3 presents a comparison between the proposed design and the most representative advanced water-cut measurement technologies reported in the literature. Unlike previous methods, the RHAMTS provides non-contact, broadband, full-range water-cut monitoring, applicable to both static and dynamic measurements. The system can be installed on storage and transportation pipelines to measure emulsified and stratified flows under realistic operating conditions, demonstrating significant practical applicability.

## 5. Conclusions

This paper presents a broadband, non-contact microwave sensing method based on the RHAMTS for water-cut monitoring in oil-water mixtures. The system operates over the 2–8 GHz frequency band and provides accurate and reliable measurements of water content in both stratified and emulsified oil-water flows. A pair of broadband ridged-horn antennas is designed and fabricated. The measured antenna reflection coefficients agree well with simulations, and the prototypes maintain S_11_ below −10 dB across most of the 2–8 GHz band, confirming good broadband matching suitable for transmission measurements. Subsequently, broadband measurements of oil-water mixtures were systematically analyzed as a function of water content. The results show that, for emulsified oil-water mixtures, both amplitude attenuation and phase shift exhibit systematic dependence on water-cut across the entire frequency band, with several frequency points showing smooth and calibration-friendly trends. Additional saline emulsion experiments further indicate that the RHAMTS can still effectively characterize water-cut under saline conditions. For stratified oil-water flow, the broadband responses remain distinguishable, and results from both static and dynamic experiments demonstrate that amplitude attenuation provides more robust features for practical water-cut discrimination. A comparison of antenna placements further demonstrates that horizontal placement provides more regular and monotonic S_21_-versus-water-cut characteristics, particularly at high water-cuts. These findings highlight the importance of frequency selection and antenna placement for robust calibration under layered flow conditions. Dynamic experiments further indicate that the RHAMTS maintains stable and monotonic response trends under flowing stratified oil-water conditions, confirming its robustness and suitability for practical pipeline monitoring. Overall, the experimental observations are consistent with the theoretical mechanism and full-wave simulations, demonstrating the feasibility of the RHAMTS as a non-contact approach for full-range water-cut monitoring. Its non-contact operation, broadband capability, and feature-selection-oriented sensing strategy provide a feasible basis for practical water-cut monitoring under complex field conditions. Future work will investigate the effects of temperature and salinity in a more systematic manner and will develop multi-frequency fusion and compensation strategies to further enhance the robustness of the system in realistic field environments.

## Figures and Tables

**Table 1:** Antenna model parameters (unit: mm).

*Wg*	*Lg*	*Lf*	*Wa*	*Hsr*	*Lsr*	*Hg*	*Sf*
21.0	17.5	60.0	108.7	0.6	10.0	14.4	2.3
*Di*	*Do*	*Sr*	*Wr*	*Wc*	*εr*	*Ha*	*θc*
1.3	4.1	0.8	5.8	1.2	2.1	46.0	45.0

**Table 2:** Parameter values of different prediction models (3.71 GHz).

Experiments	a_1_	a_2_	a_3_	R^2^
(1)	−4.185	−0.749	0.0039	0.964
(2)	−3.774	−0.759	−0.0039	0.989
(3)	−6.47	−0.66	0.0041	0.800
(4)	−7.31	−0.05	−0.0024	0.982
(5)	−5.44	−0.28	−0.0006	0.976

**Table 3:** Comparison of this work with other microwave sensors.

Works (Ref. No)	Moisture Content	Frequency Range (GHz)	Measurement Type	Applicable Flow Regimes	Operating Mode	Experiment Method
[23]	0–0.05%	9.302~9.307	Single-frequency	Dispersed flow	Non-contact	Dynamic
[24]	0.01–0.08%	10	Single-frequency	Emulsified flow	Non-contact	Static
[25]	0–100%	8.2~12.4	Broadband	Stratified flow	Non-contact	Static
[28]	0–3%	0.1–6	Broadband	Emulsified flow	Contact	Dynamic
[30]	0–100%	2–6	Broadband	Emulsified flow	Non-contact	Static
This work	0–100%	2–8	Broadband	Emulsified and stratified flow	Non-contact	Static and Dynamic

## Data Availability

The data provided in this study can be obtained from the corresponding author, due to commercial restrictions.
